# Age- and frequency-dependent changes in dynamic contrast perception in visual snow syndrome

**DOI:** 10.1186/s10194-021-01355-y

**Published:** 2021-12-11

**Authors:** Ozan E. Eren, Andreas Straube, Florian Schöberl, Ruth Ruscheweyh, Thomas Eggert, Christoph J. Schankin

**Affiliations:** 1 Department of Neurology, LMU Munich, University Hospital - Großhadern, Marchioninistraße 15, 81377 Munich, Germany; 2 Department of Neurology, Inselspital, Bern University Hospital, University of Bern, Bern, Switzerland

**Keywords:** Visual snow syndrome, Contrast threshold, Dynamic contrast perception, Visual acuity

## Abstract

**Objective:**

Patients with visual snow syndrome (VSS) suffer from a debilitating continuous (“TV noise-like”) visual disturbance. They report problems with vision at night and palinopsia despite normal visual acuity. The underlying pathophysiology of VSS is largely unknown. Currently, it is a clinical diagnosis based on the patient’s history, an objective test is not available. Here, we tested the hypothesis that patients with VSS have an increased threshold for detecting visual contrasts at particular temporal frequencies by measuring dynamic contrast detection-thresholds.

**Methods:**

Twenty patients with VSS were compared to age-, gender-, migraine- and aura-matched controls in this case-control study. Subjects were shown bars randomly tilted to the left or right, flickering at six different frequencies (15 Hz, 20 Hz, 25 Hz, 30 Hz, 35 Hz, 40 Hz). The contrast threshold (CT) for detection of left or right tilt was measured in a two-alternative adaptive forced-choice procedure (QUEST). The threshold was defined as the Michelson contrast necessary to achieve the correct response in 75% of the cases.

**Results:**

The CT increased for higher flicker frequencies (ANOVA: main effect *frequency*: F (5,180) = 942; *p* < 0.001), with an additional significant frequency*diagnosis interaction (ANOVA: F (5,180) = 5.00; *p* < 0.001). This interaction effect was due to an increased CT at a flicker frequency of 15 Hz in the VSS cohort (VSS: *MC* = 1.17%; controls: *MC* = 0.77%). At the other frequencies, group comparisons revealed no differences. Furthermore, in the VSS cohort we observed an increase of CT with higher age (r = 0.69; *p* < 0.001), which was not seen in controls (r = 0.30; *p* = 0.20).

**Conclusions:**

This study demonstrates a lower visual contrast sensitivity exclusively at 15 Hz in VSS patients and demonstrates frequency-dependent differences in dynamic contrast vision. The peak sensitivities of both parvo- and magnocellular visual pathways are close to a frequency of about 10 Hz. Therefore, this frequency seems to be of crucial importance in everyday life. Thus, it seems plausible that the impairment of contrast sensitivity at 15 Hz might be an important pathophysiological correlate of VSS. Furthermore, the overall age-related decrease in contrast sensitivity only in VSS patients underscores the vulnerability of dynamic contrast detection in VSS patients. Dynamic CT detection seems to be a promising neurophysiological test that may contribute to the diagnosis of VSS.

## Introduction

Patients with visual snow syndrome (VSS) suffer from continuous TV noise-like tiny flickering dots in the entire visual field as well as additional visual symptoms such as palinopsia and photophobia [1, 2]. The syndrome is disabling in everyday life due to the continuous presence and the lack of sufficient treatment options. In the clinical setting, the diagnosis is made solely based on the patient history since there are currently no objective measures with normal neurological and ophthalmological examinations as well as unremarkable neuroimaging. In this respect, there is no valid diagnostic test applicable that might differentiate patients with VSS from malingering [3].

Although VSS pathophysiology might at least partially overlap with migraine and particularly migraine aura, there is growing evidence that VSS comprises a distinct disorder of the visual [2, 4–6] and even extra-visual system [7]. The reported multiform visual disturbances mainly consisting of TV-noise like flickering dots, palinopsia, nyctalopia and enhanced entoptic phenomena suggest impairment of higher order visual processing. Consistently, functional imaging studies revealed hypermetabolism of brain regions important for higher order visual processing such as the lingual gyrus, but not the primary visual cortex [6, 7]. Correspondingly, a neurophysiological study brought further evidence of primarily higher order visual dysfunction in the visual association cortex as reflected by a significantly delayed N145 component of the visually evoked potentials in VSS patients [4]. In contrast, the P100 component did not differ between VSS patients and controls [4]. Nevertheless, there are also data considering the primary visual cortex or thalamocortical dysrhythmia as the underlying pathophysiological correlate of VSS [8, 9].

Regardless of the origin and underlying pathophysiology of VSS, it is still not known whether the subjective visual symptoms of VSS cause an impairment in a visual task, which can be reliably measured by a specific neurophysiological test. Despite normal daylight visual acuity, VSS patients complain of intriguing visual core symptoms, such as palinopsia, nyctalopia and TV noise-like flickering [2]. The nyctalopia and TV noise-like flickering, in particular prompt the question of whether patients with VSS exhibit different thresholds during dynamic contrast vision as compared to controls without VSS.

The aim of this study was therefore to compare dynamic contrast thresholds at different frequencies (i.e. 15, 20, 25, 30, 35 and 40 Hz) between VSS patients and control subjects that were thoroughly matched for age, sex and migraine and migraine aura, to control for the influence of migraine as suggested in previous works [6].

## Material and methods

The case-control study was conducted in accordance with the Declaration of Helsinki and approved by the local ethics committee (Nr.: 227–15). All patients gave their written informed consent. The preliminary results of the study were presented at the International Headache Conference 2019 in Dublin [10] and at the American Academy of Neurology meeting 2019 in Philadelphia [11].

### Subjects

For the recruitment of VSS patients, the study was advertised in social media with support from the self-help group for VSS, “Eye on Vision Foundation” (http://www.eyeonvision.org/). The eligibility of interested patients was assessed during telephone interviews by an VSS experienced headache specialist, which was then cross-checked by a second specialist. Later at the time of testing, the invited patients were examined by one of the two specialists involved in the telephone recruitment. All participating patients were ≥ 18 years old and fulfilled the previously published criteria of VSS (subtype black & white dots) (Schankin, Maniyar et al. 2014). The exclusion criterion was intake of illicit drugs 2 weeks prior to the onset of VSS. Patients with VSS were compared to age-, gender-, migraine- and aura-matched controls. All subjects but one per group were evaluated for depression (Patient Health Questionnaire depression scale PHQ-8) and headache frequency. Recruitment was between 2015 and 2019. Participants were compensated for travel and accommodation expanses.

### Measurement of the dynamic contrast threshold (DCTM)

During DCTM recording, participants were seated in a relaxed position in a silent room under standardized dimmed artificial light conditions. The subjects were placed at a viewing distance 60 cm in front of the screen (ASUS VG248, diagonal: 61 cm; Resolution: 1920 × 1080; 144 Hz; response time 1 ms) displaying the choice procedure. The pixel intensity of the screen was calibrated by an inverse extended power function to achieve a linear relation between the pixel intensity and the obtained luminance. Linearization was obtained between 0.2 and 110 cd/m^2^ at a resolution of 0.43 cd/m^2^. All stimuli were presented on a grey background (82 cd/m^2^). The Michelson contrast (*MC*) was defined by the ratio

$$ MC=\frac{L_{max}-{L}_{min}}{L_{max}+{L}_{min}}, $$

where *L*_*max*_ and *L*_*min*_ denote the maximum and the minimum of the luminance (cd/m^2^) of the presented pattern. Thus, the stimuli could be presented at Michelson contrasts between 5.2·10^− 3^ and 0.34.

**Fig. 1:**
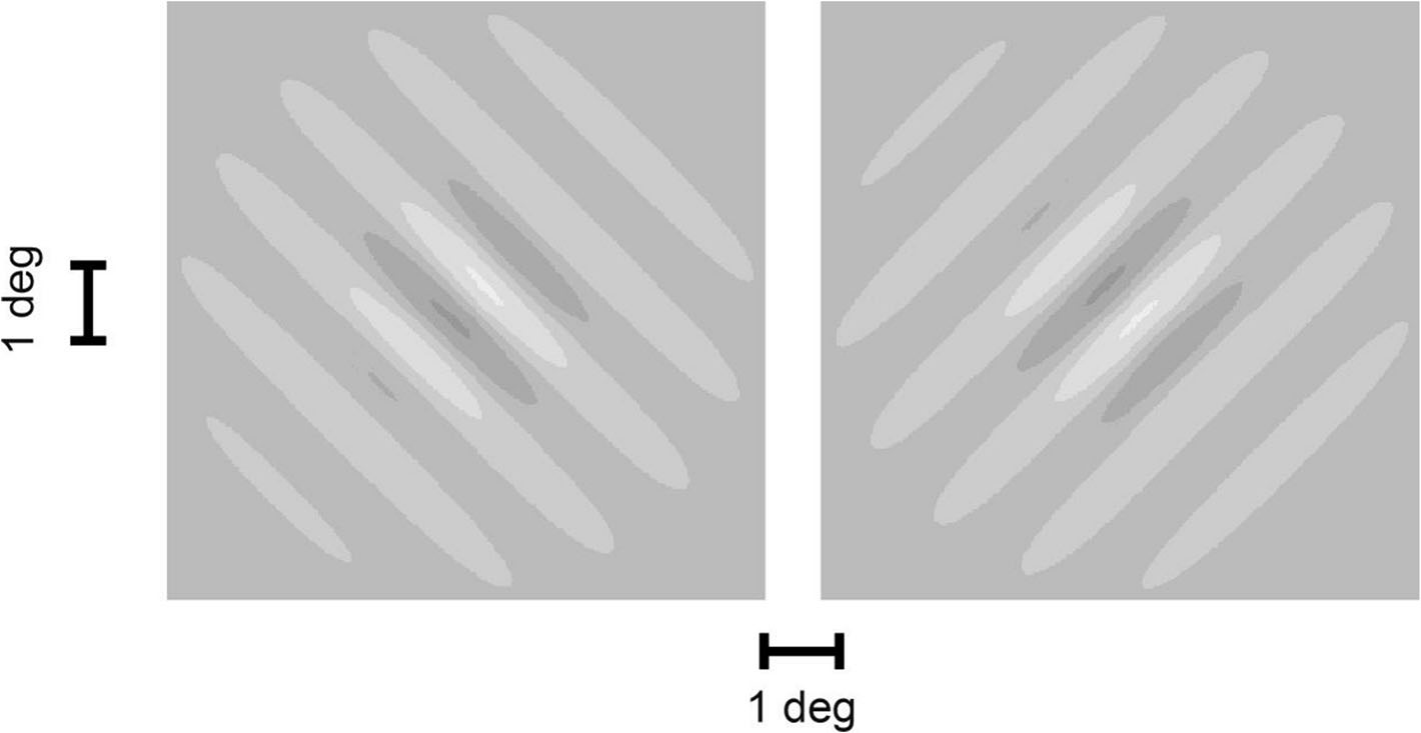
Gabor patch that served as detection stimulus. The pattern was modulated in time by a harmonic sinusoidal modulation with amplitudes between 0.37 and 28 cd/m^2^ and at six different frequencies (15 Hz, 20 Hz, 25 Hz, 30 Hz, 35 Hz and 40 Hz). Background luminance: 82 cd/m^2^. The figure shows the two alternative image orientations (leftward tilt/rightward tilt). The figures illustrate only the central portion of the screen (width: ±23.9 deg, height: ±14 deg). Outside of this window, the luminance was constant and equal to the background luminance

The subjects were instructed to focus on the fixation cross (size: 0.5 deg, luminance: 110 cd/m^2^) at the center of the screen. After pressing the spacebar, the fixation cross was replaced by a sine-grid with a spatial frequency of 0.76 cycles/deg, embedded in a Gaussian envelope with a standard deviation which was identical to the spatial period of the sine-grid (a so-called Gabor patch). The sine-grids were tilted by ±45 deg with respect to the vertical. Figure 1 shows the two versions of this Gabor patch side by side. The Gabor patch was temporally modulated at six different frequencies (15 Hz, 20 Hz, 25 Hz, 30 Hz, 35 Hz, 40 Hz). The contrast of this Gabor patch was specified as the maximum contrast during its period. After a presentation time of 1 s, the Gabor patch was replaced by the centered fixation point and the subject had to decide by pressing a button whether they perceived the grid as tilted to the right or to the left. After refixation of the centered cross, subjects pressed a button to indicate that they were ready for the presentation of the next test pattern.

The contrast threshold (CT) was measured in a two-alternative adaptive forced-choice procedure (QUEST) [12] for the discrimination between the two image orientations. This routine fitted the percentage of correct responses by a Weibull psychometric function of the log_10_ (Michelson-Contrast). For optimal efficiency, this routine iteratively adjusted the presented contrast for each individual at the level necessary to achieve 87% correct responses. At this value, the procedure is most efficient because the precision of the threshold estimate shows the fastest decrease per response. This is because the so-called sweat factor [13] of the Weibull psychometric function (lapse fraction: δ = 0.05, slope parameter: β = 3.5) adopts its minimum at that point. By controlling the obtained fraction of correct responses, we not only achieved optimal efficiency but also ensured that the task difficulty was identical for all subjects. After fitting the Weibull psychometric function to the obtained responses, the contrast threshold was defined as the Michelson contrast for which the fit predicted 75% correct responses (halfway between the 50% chance level and 100%). For each subject, and for each of the six frequencies, 80 binary responses were recorded. The choice procedure (Quest) and the stimulus presentation was controlled by using the Psychtoolbox [14] running under MATLAB (MATLAB and Statistics Toolbox Release 2014a The MathWorks, Inc., Natick, Massachusetts, United States).

### Statistical analysis

Statistical analysis was done using SPSS 25 (IBM Corp. Released 2017. IBM SPSS Statistics for Windows, Version 25.0.0.1, 32-Bit-Version, Armonk, NY: IBM Corp.).

The nominal characteristics of the groups (diagnosis, migraine) were tested for independence using the k x m contingency table [15]. All further statistics were performed on the decimal logarithm of the 75% threshold. For each group (VSS patients, controls) and for each frequency, an outlier analysis was performed to exclude extreme outliers outside the 99.9% confidence interval. Only two measurements (one control subject at a temporal frequency of 20 Hz and one VSS patient at 35 Hz) were excluded by this procedure. The Lilliefors test showed that, after this exclusion, the distribution of the log10-thresholds did not significantly differ from normal. The log-thresholds of the remaining subjects were submitted to a repeated-measures ANOVA with the group-factor *diagnosis* with two levels (control/VSS) and the within-subject factor *frequency* with six levels (15, 20, …, 40 Hz). A second ANOVA on the log-threshold was performed with the same within-factor *frequency* and the three-level group-factor *migraine* (no migraine/migraine without aura/migraine with aura). The sphericity assumption was tested by Mauchly’s test and post-hoc unpaired comparisons of the thresholds between the groups were done using unpaired t-tests separately for each frequency. The log10 (contrast threshold) was normally distributed across subjects but not the contrast threshold. Therefore, the descriptive statistics of the contrast threshold (*MC*, Table 1) are given as median [inter-quartile range].

**Table 1:** Descriptive statistics of the contrast threshold (Michelson contrast): The median and the inter-quartile range of the threshold is shown for each flicker frequency and for both groups. The last three columns show the results of the unpaired t-test of the log_10_ (contrast threshold) between patients and controls. Significant differences are marked in bold

Freq. [Hz]	Group	Contrast threshold (*MC*)	Degree of freedom	t-value	*p*-value
		median [iqr] * 100			
**15**	**controls**	**0.77 [0.61]**	**38**	**−2.83**	**0.0074**
	**patients**	**1.17 [0.51]**			
20	controls	1.68 [1.19]	37	−1.31	0.1999
	patients	1.99 [0.69]			
25	controls	2.71 [1.85]	38	−1.03	0.3088
	patients	3.10 [1.30]			
30	controls	6.08 [3.75]	38	−0.16	0.8739
	patients	6.21 [3.09]			
35	controls	7.60 [5.48]	37	−1.35	0.1841
	patients	9.12 [3.34]			
40	controls	9.29 [5.55]	38	−1.81	0.0783
	patients	11.36 [3.64]			

To investigate whether the dynamic contrast threshold depended on the migraine status in controls and VSS patients, the log-threshold was averaged across flicker frequencies for each subject and these averages were submitted to an ANOVA with the two group factors *diagnosis* (control/VSS) and *migraine status* (no migraine/migraine without aura/migraine with aura).

To understand whether there is a linear relationship between age and contrast threshold Pearson’s correlation was performed.

Statistical significance was assumed at false-positive probabilities *p* ≤ 0.05.

## Results

### Subjects

Twenty patients with VSS (7 females and 13 males, mean age 30.5 ± 10.1 years; 14 with comorbid migraine, 8 of them with typical visual aura in migraine, VSS duration 9.67 ± 10.1 years) were compared to 20 age-, gender- and migraine-matched healthy control subjects (7 females and 13 males, mean age 34.3 ± 10.4 years; 11 with comorbid migraine, 8 of them with typical visual aura in migraine). The groups did not differ in any of the mentioned categories. Submitting the frequencies of the two-level feature diagnosis (control/VSS) and the frequencies of the three-level feature migraine (no migraine/migraine without aura/migraine with aura) to a 2 × 3 contingency table showed that both features were independent of each other (χ2(2) = 1.6; *p* = 0.45). In addition, both groups showed only a low monthly headache frequency with an average of 1.37 ± 2.04 days in controls and 1.88 ± 4.67 days in VSS over the last 3 months (unpaired t-test: T (36) = − 0.436, *p* = 0.67), no subject reported headache within 48 h before and after testing. Corrected visual acuity and MRI scans (T1−/T2-weighted, Flair) were normal in all subjects. In the VSS group there was comedication with lamotrigine in one, candesartan in one and mirtazapine in three patients. There was no known long-term medication in the control group. Depression score as measured by the PHQ-8 were significantly higher in the VSS group (8.95 ± 4.85) compared to controls (1.63 ± 2.85) (unpaired t-test: T (36) = − 5.67 *p* < 0.00) but still under the cut off for major depression. Besides that, and the mentioned migraine, there was no known psychiatric or neurological disorder.

### Dynamic contrast threshold measurement (DCTM)

**Fig. 2:**
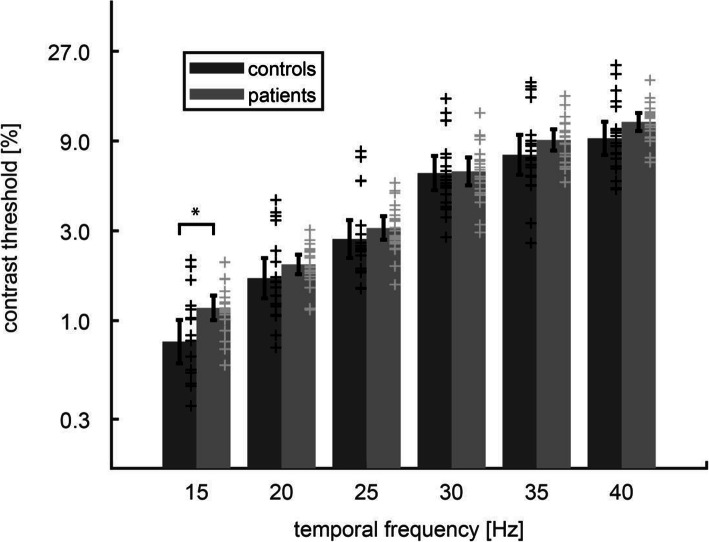
Dynamic contrast threshold as a function of temporal flicker frequency [Hz] for VSS patients and controls. Bars and whiskers show the mean of the log10 (contrast threshold) and the 95% confidence interval of the mean. Each cross indicates the threshold of a single subject. VSS patients showed higher contrast thresholds at 15 Hz (asterisk, *p* < 0.01) but did not differ from controls at higher frequencies

Dependence of the contrast threshold on the flicker frequency is shown for VSS patients and controls in Fig. 2. The threshold increased with increasing flicker frequency (ANOVA: main effect *frequency*: F (5,180) = 942; *p* < 0.001). In the absence of a main effect for the factor *diagnosis* (F (1, 36) = 1.90; *p* = 0.18), the interaction effect was significant (frequency*diagnosis: F (5,180) = 5.00; *p* < 0.001). This interaction effect was because VSS patients showed an increase of contrast threshold (*MC* = 1.17%) compared to the control group (*MC* = 0.77%) (*p* = 0.0074) at the specific flicker frequency of 15 Hz. At all the other frequencies (i.e. 20, 25, 30, 35 and 40 Hz), contrast thresholds did not differ between VSS and controls (*p* < 0.05) (Table 1).

The migraine status (no migraine/migraine without aura/migraine with aura) did not affect the contrast thresholds at any frequency since there was no significant main effect or interaction in the ANOVA for the factor *migraine* (*p* > 0.12). The median of the contrast threshold across all subjects was 3.72 [iqr = 1.90] %.

**Fig. 3:**
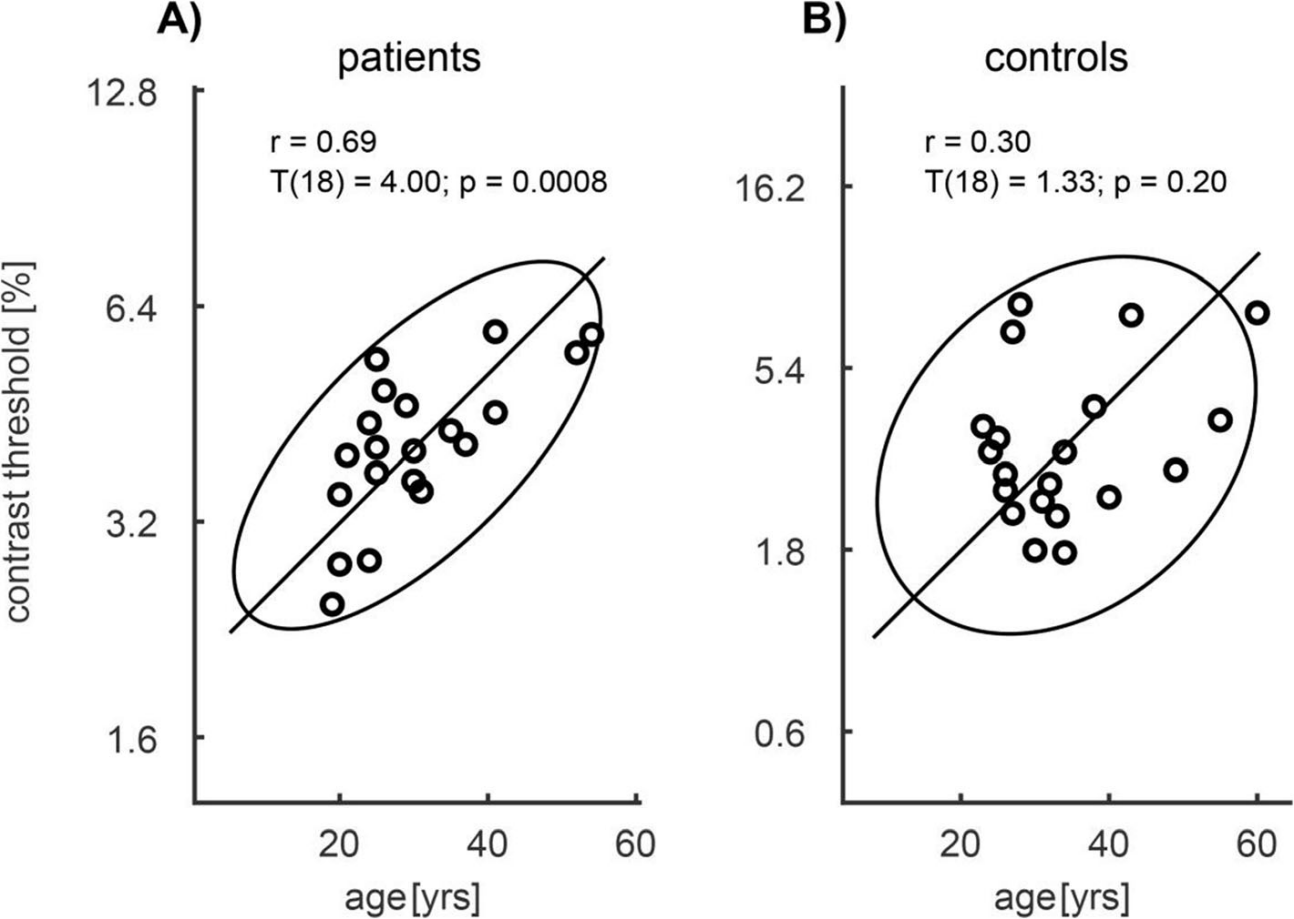
Dependence of the contrast threshold in controls (**A**) and VSS patients (**B**) on age. Solid: the 95% confidence ellipse and its great semi axis. r: Pearson’s coefficient of correlation between age and contrast threshold. Significant correlation was observed in patients (*p* < 0.001) but not in controls

In the VSS cohort, we found a significant positive correlation of the log-contrast threshold with age across all frequencies (r = 0.69; *p* < 0.001) (Fig. 3 A), which did not occur in the control group (r = 0.30; *p* = 0.20) (Fig. 3 B). This increase of the threshold with increasing age in the patient group was not due to disease duration since the correlation between the log-contrast threshold and the disease duration was non-significant (r = 0.19; *p* = 0.46).

Using the contrast threshold at 15 Hz for discriminating between patients and controls leads in the ROC analysis to a correct decision rate of 0.725 with a sensitivity of 1.0 and a specificity of 0.45.

## Discussion

The main finding of this study is that VSS patients showed an impaired contrast threshold for temporal modulation frequencies of 15 Hz and the spatial frequency of 0.76 deg/s. For higher frequencies, the contrast thresholds were not significantly affected. For the 15 Hz stimulus, the observed contrast threshold of our control subjects (0.77%) corresponded to a contrast sensitivity of 130 (=100/0.77), which is similar to values reported in the literature [16].

There is not much research on this topic with which to compare our study, although there have been first attempts at psychometric measurements using visual tasks in VSS before. It is important to consider that visual tasks can be programmed very differently as there is no standard procedure, which is why the exact parameters are mentioned for spatial and temporal frequencies whenever necessary. McKendrick and colleagues [17], for example, measured only a single spatial frequency (2 cycle/deg) at constant presentation (temporal frequency 0 Hz) and also found an impairment of VSS patients regarding contrast sensitivity despite a different approach. Our study shows that this impairment is specific for the lower range of temporal frequencies. Thus, the question arises whether the specificity of the impairment to a low range of temporal frequencies may provide hints as to the location of the functional impairment within the visual system.

Recent functional and structural imaging data pointed towards relevant changes in associative visual brain regions beyond the primary visual cortex that would be important for higher order visual processing such as the lingual gyrus [2, 7, 18, 19]. This is supported by electrophysiological data of a normal P100-, but significantly delayed later N145-component of the visual evoked potentials in VSS patients, thus implying a dysfunction of higher visual processing in extrastriate brain regions downstream from the primary visual cortex [4]. However, it remains unclear why patients with VSS suffer from such intriguing visual symptoms as palinopsia, nyctalopia and particularly TV noise-like flickering in the absence of daylight visual acuity impairments. Therefore, an approach from a neurophysiological perspective seems reasonable. The visual system (from the retina to the occipital visual areas) can be divided into two major visual pathways: the predominant parvocellular and the less populated magnocellular pathway [20]. Both systems serve partially different visual domains but also show some overlap [21]. Physiologically, the magnocellular pathway is less color-dependent but shows a higher contrast sensitivity than the parvocellular pathway. In addition, the magnocellular pathway is activated by lower spatial and higher temporal frequencies, whereas the opposite seems to be the case for the parvocellular pathway [22–25].

Skottun and Skoyles [26] tried to answer the proportion of the parvo- and magnocellular pathway responding to different temporal frequencies and found that a frequency of 10 Hz is similarly effective in stimulating both systems. To stimulate the magnocellular pathway activation alone, temporal frequencies well above 20 Hz are required [26], a frequency at which our patients were unimpaired. Despite the large overlap between the parvo- and magnocellular systems in the temporal frequency domain, our results point towards dysfunction of the parvocellular, rather than the magnocellular pathway [26].

Such involvement of the parvocellular system is supported by the significant positive correlation of dynamic contrast thresholds and age only in patients with VSS, but not in healthy subjects (Owsley and Sloane 1987). Such age-related clinical worsening has not been reported in VSS so far, although progressive forms have been described [2]. One might speculate that, at least from a psychophysiological perspective, VSS could be a progressive disorder. One explanation could be that the parvocellular system, which involves more color perception neurons with high energy demand as shown by the mitochondrial density, may be more vulnerable to the presumable continuous neuronal hyperactivity present in these patients. Fittingly, correlates of such hyperactivity with grey matter alterations have been demonstrated in functional and structural neuroimaging in VSS in the medial temporal lobe [7], which is part of the ventral stream of cortical visual processing, fed primarily by the parvocellular system.

Regarding the spatial frequency, we tested at a specific range (0.76 cycles/deg) without any modulation. Impairment at a low temporal and spatial frequency, as detected in the present study, is not at odds with the fact that VSS patients have normal static visual acuity [2, 4, 5]. Static visual acuity depends mainly on the highest spatial frequencies [27] and is only marginally affected by the removal of low spatial frequencies.

At the end you may ask is if it is possible to discriminate between patients and controls using the contrast threshold at 15 Hz. The ROC analysis for an estimated correct decision rate of 0.725 showed a sensitivity of 1.0 and just a specificity of 0.45, which shows that CT measurement in its current form can be a supportive but not the only used tool in the diagnostic of VS patients.

### Limitations

The main limitation of the study is that lower temporal frequencies were not studied, as there had to be a compromise between the total duration of a demanding setting and the region of interest regarding lower and higher frequencies. In future research the focus should be shifted to even lower temporal frequencies as our data suggests that higher temporal frequencies are not impaired. Additionally, there is always the chance of a potential selection bias towards more affected subjects as the first contact was mainly established through the mentioned self-help group for VSS. On the other hand, we carefully reexamined our patients by two experienced investigators. Depression scores were higher in VSS compared to controls as also known from many other chronic neurological disorders [28]. However, VSS patients were highly motivated, the study protocol itself did not depend on speed and pauses were possible if needed. Importantly we decided to match both groups for migraine with and without aura. The intention of not investigating against a control group with only healthy subjects was to directly control for migraine and migraine aura. A control group with only healthy subjects might have resulted in comorbid migraine in VSS confounding the results, especially since it has been shown that migraine is associated with alterations in contrast threshold [29, 30], however that was not the case in our study as migraine status did not affect the contrast thresholds at any frequency. To control also for the migraine cycle [31], both groups were measured interictally. However, future studies would be necessary to compare VSS without any comorbidity, especially migraine, migraine aura and depression to healthy controls to confirm our findings.

## Conclusion

This study gives new insights into visual snow pathophysiology because it demonstrates two points: First, there is an isolated difference in dynamic visual contrast sensitivity in VSS at lower frequencies (15 Hz) compared to gender-, age-, migraine-, and aura-matched controls. The difference at 15 Hz is near the optimum frequency of both major visual processing streams, the parvo- and magnocellular pathways.

Secondly, VSS patients showed a positive correlation of age and lower contrast sensitivity, which was not seen in matched controls.

Dynamic contrast threshold detection seems to be a promising neurophysiological test that may contribute to our understanding of VSS.

As already mentioned in the limitations, future research should focus on lower temporal frequencies, additionally a longitudinal examination of VSS patients has to be performed in order to understand long-term or possible adaptation effects.

## Data Availability

Anonymized data will be shared by request from any qualified investigator.
